# Draft Genome Sequence of *Xylaria hypoxylon* DSM 108379, a Ubiquitous Fungus on Hardwood

**DOI:** 10.1128/MRA.00845-19

**Published:** 2019-10-31

**Authors:** Enrico Büttner, Christiane Liers, Martin Hofrichter, Anna Maria Gebauer, Harald Kellner

**Affiliations:** a Department of Bio- and Environmental Sciences, International Institute Zittau, Technische Universität Dresden, Zittau, Germany; University of California, Riverside

## Abstract

The saprotrophic ascomycete Xylaria hypoxylon is a widespread wood-decaying fungus on deciduous trees. Here, we report its draft genome sequence. The genome assembly has a size of 42.8 Mbp and a G+C content of 47.1% and includes 11,038 predicted genes.

## ANNOUNCEMENT

Xylaria hypoxylon (carbon antlers) is the type species of the genus Xylaria already described by Carl Linnaeus (1, 2). It is very common on tree stumps or woody material of broad-leaved trees buried in the soil and is widely distributed in northern temperate Europe and western North America (1). In fruiting bodies and cultures of this fungus, several secondary metabolites and proteins with potential for application in biotechnology or medicine have been found (3). Thus, the structures of succinic acid derivatives (4), melleins (5), xylarone and its derivatives (6), and new tetralone derivatives (7) were elucidated and showed promising biological activities. Furthermore, lectins with hemagglutinating, antiproliferative, and antimitogenic activities were found (8). Moreover, it was shown that X. hypoxylon is able to degrade and mineralize lignin to some extent in lignocellulose-based solid-state cultures (9).

Overall, the presented genome can help in identifying enzymes of ecological and biotechnological relevance, in assisting the identification of biosynthetic clusters, and in comparing genomic data of the important ascomycetous order Xylariales.

*X. hypoxylon* DSM 108379 (ribosomal cistron; GenBank accession number MK577428) was collected on rotting stumps of Fagus sylvatica (50°24′12.5″N, 11°32′58.2″E, Jägersruh-Gemäßgrund-Mulschwitzen, Bad Lobenstein, Germany). The fungus was cultured in liquid whey-protein glucose medium (2.5%) for biomass production. Genomic DNA was extracted using a standard cetyltrimethylammonium bromide (CTAB)-based protocol. Genome sequencing was performed using the Ion Torrent Personal Genome Machine (PGM) platform (Ion PGM sequencing 200 kit version 2, 318 v2 Chip, and Ion Xpress Plus 200-bp fragment library kit; Thermo Fisher, Darmstadt, Germany). The resulting reads were filtered for quality, using Geneious R11 (trim 3′ end; error probability limit, 0.05) (10) and length (160- to 270-bp reads were included). A total of 4.7 million reads of an average of 230 bp were *de novo* assembled using MIRA 4.0 (11), and a second step with Geneious R11 was used to filter out duplicate contigs. The assembly consists of 635 contigs with a total length of 42.8 Mbp (maximum contig size, 582,987 bp). The assembly was verified using QUAST v4.5 (12) and has an *N*_50_ value of 122,761 bp and a G+C content of 47.1%. The completeness of the assembly was assessed using BUSCO v3 (predictor, Aspergillus nidulans; fungal data set, Ascomycota_*odb9*) and has a completeness of 96.0% (13). Gene prediction was performed using AUGUSTUS v3.2.2 (predictor, Aspergillus nidulans) (14) and resulted in 11,038 protein-coding genes. Genes were annotated with Blast2GO v5.2.2 (BioBam, Valencia, Spain) and dbCAN (HMMdb v7; E value < 1E^−15^, coverage > 0.35) (15). Altogether, 678 carbohydrate-related enzymes and modules (among them were 140 enzymes with auxiliary activity) were identified.

Enzymes involved in the oxidative degradation of lignocellulose and the conversion of aromatics such as cellobiose dehydrogenase, laccases/oxidases, dye-decolorizing peroxidases, and heme-thiolate peroxidases/peroxygenases were manually annotated and are available in GenPept (accession numbers shown in Table 1). Secondary metabolite biosynthetic gene clusters (BGCs) were predicted using antiSMASH v4.1.0 (16). A total of 53 BGCs were identified, including BGCs for the production of 27 polyketides, 19 nonribosomal peptides, and seven terpenes.

**TABLE 1:** CAZyme classes and enzymes of interest detected in the genome of DSM 108379

Enzyme or domain group	No. of proteins	GenPept accession no.
Glycoside hydrolases	269	
Glycosyl transferases	95	
Polysaccharide lyases	16	
Carbohydrate esterases	111	
Auxiliary activity	140	
Associated modules		
Carbohydrate-binding modules	47	
Cellulose-binding domain CBM1	12	
Enzymes of interest		
Unspecific peroxygenase	3	TGJ83873, TGJ80919, and TGJ78181
Laccase	10	TGJ88672, TGJ88079, TGJ87255, TGJ87003, TGJ85732, TGJ83324, TGJ82763, TGJ82599, TGJ79719, and TGJ78816
Generic peroxidase (class II related)	2	TGJ80457 and TGJ79548
Dye-decolorizing peroxidase	3	TGJ80762, TGJ84175, and TGJ88548
Cellobiose dehydrogenase	1	TGJ88735

### Data availability.

This whole-genome shotgun project has been deposited at DDBJ/ENA/GenBank under the accession number SKBN00000000. The version described in this paper is the first version, SKBN01000000. The Sequence Read Archive (SRA) accession number is SRR8662833. The associated BioProject accession number is PRJNA525368.
